# Cyclic Cushing’s syndrome in ACTH-dependent hypercortisolism induced by the immune checkpoint inhibitor pembrolizumab

**DOI:** 10.20945/2359-4292-2024-0435

**Published:** 2025-09-28

**Authors:** Laura Borja Pardini, Ingrid Silva Bremer de Toledo, Aline Ramos Amaral, Vitória Donadoni Costa, Pedro Weslley Souza do Rosário

**Affiliations:** 1 Departamento de Endocrinologia e Metabolismo, Santa Casa de Belo Horizonte, Belo Horizonte, MG, Brasil; 2 Universidade Federal de Minas Gerais, Belo Horizonte, MG, Brasil

**Keywords:** Cushing’s syndrome, immune checkpoint inhibitors, cyclic hypercortisolism

## Abstract

Immune checkpoint inhibitors have become transformative therapies, significantly enhancing survival outcomes for various neoplasms. However, they often trigger immune-related adverse events, including endocrinopathies. Cushing’s syndrome, characterized by exposure to elevated levels of circulating cortisol, presents a wide range of clinical features and is closely associated with increased morbidity and mortality. This article reports on a case of a patient under checkpoint inhibitor therapy, who developed cyclic adrenocorticotropic hormone-dependent hypercortisolism. The patient exhibited a Cushingoid phenotype, and testing revealed increased cortisol levels following the administration of 1 mg of dexamethasone, indicating endogenous hypercortisolism. Notably, the cortisol levels followed a cyclic pattern, decreasing as the next dose of pembrolizumab neared, thereby linking the hypercortisolism to fluctuations in the medication’s serum concentration. Given the significant morbidity linked to hypercortisolism, it is crucial for physicians prescribing immune checkpoint inhibitors to recognize the potential onset of endocrinopathies with unconventional presentations, such as cyclic hypercortisolism. Such conditions may present diagnostic and therapeutic challenges, ultimately impacting patient survival.

## INTRODUCTION

Immune checkpoint inhibitors represent a promising therapeutic avenue that may enhance survival rates across various neoplasms. Although endocrinopathies such as thyroid disorders and hypopituitarism, secondary to hypophysitis, have been noted as autoimmune adverse effects, lesions affecting the gastrointestinal, pulmonary, renal, and skin systems are more frequently observed (^1^,^2^). Endogenous Cushing’s syndrome, an endocrinopathy induced by excessive levels of circulating cortisol, is characterized by a spectrum of clinical manifestations that contribute to increased morbidity and mortality (^3^). This article reports on a case in which a patient undergoing therapy with immune checkpoint inhibitors for cancer developed Cushing’s syndrome due to adrenocorticotropic hormone (ACTH)-dependent cyclic hypercortisolism.

## CASE PRESENTATION

A 28-year-old woman, previously healthy, was diagnosed with stage IIID melanoma. The initial lesion was located on the right leg, with subsequent metastasis to the femoral lymph node, lung, optic tract, and subcutaneous nodules. As a second-line therapy, pembrolizumab 200 mg was administered every three weeks, completing the first cycle. Concurrently, the patient underwent stereotactic radiotherapy for the optic tract lesion, and high doses of dexamethasone were administered for neuroprotection over 30 days, followed by a gradual decrease over the next 30 days.

The day after the second pembrolizumab infusion, hospitalization was required. The patient exhibited a Cushingoid phenotype, acne, secondary amenorrhea, proximal muscle weakness, and weight gain – symptoms initially considered to result from the prior dexamethasone regimen, which had been discontinued 10 days before hospitalization. In light of these symptoms and a slightly elevated basal cortisol level (8 AM cortisol = 23.7 mcg/dL), an investigation for Cushing’s syndrome commenced. With normal liver and kidney function and no use of medications known to affect dexamethasone metabolism or increase corticosteroid-binding globulin levels, a cortisol assay post 1 mg dexamethasone administration was conducted. The test was properly conducted, revealing unsuppressed cortisol levels (18.1 mcg/dL). In the hospital setting, a midnight serum cortisol assay confirmed endogenous hypercortisolism (9.9 mcg/dL). This result is consistent with research indicating that midnight serum cortisol levels greater than 7.5 mcg/dL have sensitivity and specificity exceeding 96% (^4^).

Continuing the diagnostic process, basal ACTH levels were measured. The initial ACTH level was indeterminate (16.6 pg/mL) but later escalated to 42.8 pg/mL, confirming the ACTH-dependent hypercortisolism (^5^). Subsequent basal cortisol levels, following an 8 mg dexamethasone suppression test, were 16.5 mcg/dL and 2.5 mcg/dL, respectively (**Table 1**). The 84% reduction in cortisol levels confirms a diagnosis of ACTH-dependent hypercortisolism of pituitary origin, corroborated by studies indicating that a serum cortisol reduction greater than 75% in the 8 mg dexamethasone suppression test has a 100% specificity for this diagnosis (^5^). A pituitary magnetic resonance imaging (MRI) ruled out pituitary lesions, showing homogeneous parenchyma, regular contours, and no signal abnormalities. The optic pathway lesion previously identified remained unchanged.

**Table 1:** Hypothalamic-pituitary-adrenal axis after pembrolizumab administration

Variables	28/03/22	29/03/22	06/04/22	13/04/22	14/04/22	20/04/22	21/04/22	24/04/22	26/04/22	27/04/22	03/05/22	04/05/22
Cortisol 8 AM (3.7–19.4) (mcg/dL)	PA	23.7		16.5			16.3	PA	14.7	17.9	11.3	
Midnight serum cortisol (mcg/dL)				9.9								
ACTH (normal range < 46) (pg/mL)				16.6			32.6		22.5	42.8	21.6	
Low-dose dexamethasone suppression test (mcg/dL)			18			16.1						23.4
High-dose dexamethasone suppression test (mcg/dL)					2.50							

ACTH: adrenocorticotropic hormone; PA: pembrolizumab administration.

**Figure 1:**
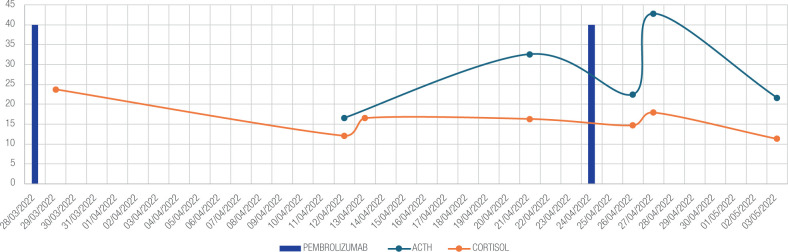
Fluctuations in ACTH and serum cortisol levels in relation to the timing of Pembrolizumab administration

During follow-up, cortisol and ACTH levels showed significant fluctuations, especially immediately following pembrolizumab administration, when levels peaked and then gradually decreased as the date for the next dose neared. Further evaluation of the hypothalamic-pituitary axis post-medication administration revealed increases in other pituitary hormones, including growth hormone (5.4 ng/mL) and prolactin (40.27 ng/mL) (**Table 2**). Following the third pembrolizumab cycle, elevated ACTH and cortisol levels remained, despite a 1 mg dexamethasone suppression test (cortisol = 23.4 mcg/dL; basal ACTH = 21.6 pg/mL), but showed a gradual decline over time (**Figure 1**).

**Table 2:** Other hormones assessed during follow up after pembrolizumab administration

Variables	21/04/22	24/04/22	27/04/22	03/05/22
LH (normal range 1.8-11.78) (mIU/mL)	1.09	PA	1.03	0.94
FSH (normal range 3.03-8.08) (mIU/mL)	3.74		4.3	3.48
Oestradiol (normal range 21-251) (pg/mL)	20		21	28
Prolactin (normal range 5.18-26.53) (ng/mL)	47.53		40.27	33.72
TSH (normal range 0.35-4.94) (mcIU/mL)	9.37			
Free T4 (normal range 0.7-1.48) (ng/dL)	1.01			
GH (normal range < 8) (ng/mL)	5.4		13.5	3.08
IGF-1 (normal range for ages 83-259) (ng/mL)	556		52	41.6

LH: luteinizing hormone; FSH: follicle-stimulating hormone; TSH: thyroid-stimulating hormone; GH: growth hormone; IGF-1: insulin-like growth factor 1; PA: pembrolizumab administration.

Given the advanced stage of the primary lesion and the mild hypercortisolism, the decision was made not to discontinue pembrolizumab and to continue monitoring the patient. However, on the day scheduled for the fourth cycle of the medication, the patient’s clinical condition deteriorated, leading to infectious complications and the eventual interruption of clinical follow-up.

## DISCUSSION

Our team reports the second documented case of ACTH-dependent hypercortisolism associated with pembrolizumab. The first case was published in 2022 by a French team. The case described by Paepegaey and cols. (^6^) shares significant similarities with ours. In both instances, confirmatory tests indicated that the hypercortisolism was ACTH-dependent, and pituitary MRIs, which showed no evidence of pituitary lesions, were conducted. Notably, cyclical hypercortisolism was observed, with increases in free urinary cortisol and ACTH levels a few days following pembrolizumab administrations.

A comprehensive review by Tan and cols. (^7^) discussed endocrine alterations linked to immune checkpoint inhibitors. The earliest case of ACTH-dependent hypercortisolism followed by adrenal insufficiency, following the use of ipilimumab combined with nivolumab, was reported in 2017 by Lupu and cols. (^8^). This report marked the first instance of ACTH-dependent hypercortisolism related to immune checkpoint inhibitors. AlRubaish and cols. (^9^) recently documented a case of transient ACTH-dependent hypercortisolism associated with nivolumab-ipilimumab in 2024, which bears striking resemblance to the one we are discussing. Post-immunotherapy initiation, the patient was diagnosed with ACTH-dependent hypercortisolism. Similar to our case, the patient exhibited additional functional pituitary alterations, including central hypothyroidism and hypogonadotropic hypogonadism, with normal adrenal and pituitary imaging findings. Crucially, all these cases demonstrated cortisol secretion cycles dependent on the timing of the medication administration (^7^).

Our case underscores significant fluctuations in cortisol and ACTH levels following each Pembrolizumab cycle, with levels peaking in the days immediately following medication administration and gradually declining as the next dose approached. The secondary hypophysitis induced by immune checkpoint inhibitors offers a plausible explanation, recognized as a common adverse event associated with these medications, typically presenting with nondescript pituitary MRI findings (^10^). However, our case is distinguished by its documentation of both clinical and laboratory features of hypercortisolism associated with immune checkpoint inhibitor use, a phenomenon sparsely reported in literature.

Hypophysitis triggered by immune checkpoint inhibitors is typically characterized by a destructive process often leading to hypopituitarism and adrenal insufficiency. The presence of preceding hypercortisolism, as in this case, is exceptionally rare and seldom reported. Uniquely, this patient exhibited cyclic elevations in serum ACTH and cortisol levels following each pembrolizumab cycle.

Due to the patient’s clinical deterioration and subsequent death from infectious complications, long-term follow-up to evaluate potential hypopituitarism development was not feasible. Nonetheless, it is theorized that other, yet to be delineated, pathophysiological mechanisms contributed to the patient’s presentation. One plausible mechanism involves an “activating” autoimmune impact on certain cellular lineages, such as corticotroph cells, potentially elucidating the hormonal fluctuations noted in correlation with the medication’s bodily concentration.

Immune checkpoint inhibitors’ mechanism of action can activate self-reactive T cells, precipitating specific immune-related adverse events akin to autoimmune diseases (^6^). Hormonal alterations linked to these medications’ use are increasingly reported as their application in treating various neoplasms expands, proving highly effective in enhancing survival rate (^8^). Nevertheless, while endocrine alterations are documented, their exact incidence remains uncertain (^11^). Risk factors predisposing individuals to these adverse reactions remain undefined (^1^,^3^). Cushing’s syndrome is a rarely reported, yet significant, adverse effect of checkpoint inhibitor. However, morbidity related to hypercortisolism is significant, particularly in patients in clinically fragile conditions. In our case, the patient succumbed to a pulmonary infection weeks after initiating endocrine monitoring.

Given our patient’s swift clinical decline, determining the full impact of ACTH-dependent hypercortisolism and its contribution to her prognosis is challenging. The risk of endocrinopathies associated with immune checkpoint inhibitors is well-documented (^7^); however, Cushing’s syndrome remains rare and potentially severe. It is recommended that when treating a patient with immune checkpoint inhibitors, the attending physician should remain vigilant for the emergence of endocrinopathies that can jeopardize patient survival. Additionally, the physician should be attentive to electrolyte disturbances, weight gain, proximal muscle weakness, and other symptoms indicative of hypercortisolism, which would enable early diagnosis and appropriate intervention.

## Data Availability

datasets related to this article will be available upon request to the corresponding author.
